# Owners’ Attitudes toward Their Companion Dogs Are Associated with the Owners’ Depression Symptoms—An Exploratory Study in South Korea

**DOI:** 10.3390/ijerph16193567

**Published:** 2019-09-24

**Authors:** Kyung-Duk Min, Woo-Hyun Kim, Seongbeom Cho, Sung-il Cho

**Affiliations:** 1 Department of Public Health Science, Graduate School of Public Health, Seoul National University, Seoul 08826, Korea; kdmin11@hotmail.com; 2 Research Institute for Veterinary Science and College of Veterinary Medicine, Seoul National University, Seoul 08826, Korea; iceman2b@snu.ac.kr; 3 Institute of Health and Environment, Seoul National University, Seoul 08826, Korea

**Keywords:** Zooeyia, modified Pet Attitude Scale, companion dogs, depression

## Abstract

*Background:* Various health benefits from the ownership of companion dogs have been studied from a One Health perspective. However, the preventive effects on depression are unclear, with inconsistent results across studies. We hypothesized that heterogeneity among owners would be related to the mixed results. Specifically, the difference in the strength of the bond between the owners and their companion dogs would modify the effect of dog ownership. As an exploratory study, we compared the depression symptoms of the owners with favorable attitudes toward their dogs, with those of the owners with unfavorable attitudes, to investigate the potential effect modification of owners’ attitudes on the association between the ownership and depression symptom. *Methods:* We conducted a web-based questionnaire survey of 654 19- to 39-year-old adults who had companion dogs in Seoul, South Korea, where a major health burden is depression among young adults. We measured the owners’ attitudes toward their dogs using the modified Pet Attitude Scale (PAS-M) and their depression symptoms using the short version of the Center for Epidemiologic Studies depression scale (CESD-10). Demographic and socioeconomic factors were measured to adjust for the association between attitude and depression symptoms. Multivariate logistic regression models were used in this study. *Results:* The owners who had less favorable attitudes toward their dogs (lower PAS-M scores) tended to have depression symptoms. The direction and significance were maintained either when the PAS-M variable was used as a continuous variable (odds ratio (OR) for one score increase in PAS-M was 0.95 (95% confidence interval (CI) = 0.94–0.96)) or as a categorical variable (OR for lower PAS-M was 3.19 (95% CI = 2.28 –4.47)). *Conclusion:* We found a positive significant association between owners’ depression symptoms and unfavorable attitudes toward their dogs, although causal direction could not be determined. Future studies should investigate the potential causal link.

## 1. Introduction 

Zooeyia is a One Health approach that focuses on the interface between companion animals and human health [1]. Using the concepts of zooeyia, health benefits of companion dogs either through ownership or animal-assisted therapy (AAT) have been studied. The studies often focused on companion dogs possibly due to their popularity in both ownership and AAT. For example, a positive association between physical activity and dog ownership has been reported in both adolescents [2] and adults [3], and an American Heart Association review suggested potential preventive effects on cardiovascular disease [4]. In addition, the association of dog ownership with increased social capital [5,6,7] and well-being [8] has been reported, and many other potential health benefits, including increased quality of life, reduced loneliness, and positive psychosocial development in children have also been suggested [9,10].

The benefits of companion dog ownership on mental health have been reported in various populations [11], including adolescents [12], street-involved youth [13], patients living with human immunodeficiency virus [14], and patients with mental health problems [15]. However, several studies found that the association was not conclusive [16,17,18,19]. Methodological limitations, such as cross-sectional study designs, could cause the inconsistent findings [9,20], but sociodemographic differences among the owners and their effect modification [21], or variations in health status of the dogs [22] can also be explanations. 

Meanwhile, heterogeneity among the owners, specifically, the strength of the human–animal bond could be another explanation. Curry et al. [23] found that the people who have owned companion dogs previously tend to produce more oxytocin, than those who have not, during interaction with companion dogs. Considering that oxytocin was suggested as a mediator of the health benefit [24], and previous exposures to companion animals is associated with favorable attitude toward companion animals [25], the owners’ attitude could be associated with the health benefit of dog ownership. In other words, the health benefit could be diluted in owners who have unfavorable attitudes toward their dogs.

In this study, we explored the hypothesis that the strength of the human–animal bond affects the mental health benefits of dog ownership by examining the association between owners’ attitudes toward their dogs and the owners’ depression symptoms. Although a prospective longitudinal study is necessary to reveal causal associations, we used a cross-sectional design with a relatively small sample size as an exploratory study. The study population was limited to young adults aged between 19 and 39 to minimize heterogeneity by various age groups. Specifically, we excluded older adults due to the possible confounding effects of their comorbid chronic diseases, and younger children were not included because of their parental dependency. Participants who aged 40 or more were not considered because their health burden of depression was relatively minor in South Korea [26], where the study was conducted. 

## 2. Materials and Methods 

### 2.1. Web-Based Survey Implementation

An on-line survey was conducted from September to October in 2017, in collaboration with Research and Research (Seoul, South Korea), which operates a prebuilt panel group, called the R panel (http://panel.randr.co.kr/), consisting of approximately 1.13 million South Koreans over 14 years old. We randomly emailed 25,000 registered panel group members and 654 ultimately completed the survey. In this process, we limited the participants to young adults between 19 and 39 years old who were currently living with companion dogs in Seoul. People who raised dogs for commercial purposes were excluded.

### 2.2. Questionnaire Development 

The questionnaire included questions about nine factors: depression symptoms, owners’ attitudes toward their dogs, age, sex, education, income level, marital status, employment, and family size (Table 1). To examine the owners’ depression symptoms, which was the main health outcome of this study, we used the short version of the Center for Epidemiologic Studies Depression scale (CESD-10) [27]. The CESD-10 is comprised of 10 questions related to the subjects’ feelings and behaviors in the last 7 days. Each question has four response options indicating the frequency of the feelings and behaviors, and was scored from 0 to 3. We defined owners as having depression symptoms if their total scores exceeded 10, as suggested in Andresen et al. [27]. We used the Korean version, as used in the Korean Longitudinal Study of Ageing [28]. The modified Pet Attitude Scale (PAS-M) [29] was used to capture the owners’ attitudes toward their dogs, which was the main explanatory variable in this study. They coined the term “pet” for the indicator because the measure was first developed in 1981 [30]. The PAS-M consists of 18 questions with answers on a 7-point Likert scale (Table 2). Each question was scored from 1 to 7 (6 of the 18 questions are reverse score items), and a higher sum of the scores indicated more favorable attitudes toward companion animals [31] (the range of the possible scores is from 18 to 126). The original English version was translated by two researchers using a forward–backward translation procedure [32], as there was no Korean version of the PAS-M. The questions on demographic characteristics and socioeconomic status were adopted from items in the Korean National Health and Nutritional Examination Survey [33] (Table 1). 

The Institutional Review Board (IRB) at Seoul National University granted exemption for this study (IRB approval number: SNU IRB No. E1708/003-001).

### 2.3. Statistical Analysis 

Descriptive analysis was used to overview the participants’ sociodemographic and economic status, such as age, sex, education attained, household income, marital status, employment, and family size. The prevalence of the participants’ depression symptoms and average PAS-M score were also explored. Stratifying the study population into those with and without depression symptoms, we also examined the general characteristics of each group. Pearson’s chi-square test and the student’s t-test were used to identify significant differences in the characteristics of categorical and continuous variables in the two groups, respectively.

The association between owners’ depression symptoms and PAS-M adjusted by covariates, including demographic and socioeconomic factors, was investigated using multivariate logistic regression models. Two logistic regression models were used to check the robustness of the association: Model 1 used the PAS-M score as a continuous variable and Model 2 included it as a dichotomous variable using the median value as a cut-off. Multicollinearity was checked using generalized variance inflation factors (GVIF) [34] because the models included categorical variables. We excluded covariates with GVIF^1/(2*df)^ > 2, which were considered variables with high correlations, following previous studies [35,36,37]. The odds ratio (OR) and 95% confidence interval (95% CI) were used to show the effect size and statistical significance. We also conducted sensitivity analysis using the depression symptoms variable as the total score of the CESD-10 (a continuous variable) (Models 3 and 4). General linear models were used to this end. All statistical analyses were conducted using R ver. 3.5.1.

## 3. Results

### 3.1. General Characteristics of Study Participants

Table 3 summarizes the general characteristics of the 654 study participants: A total of 470 (71.9%) participants were between 30 and 39 years old; 362 (55.4%) were male; 556 (86.5%) graduated from university; 324 (49.5%) were living with spouses; while 31 (4.7%) were living alone; 568 (86.9%) were employed; 320 (48.9%) were current smokers; 624 (95.4%) were current drinkers; 19.1% exercised regularly; and 353 (54%) had depression symptoms.

The differences in demographic and socioeconomic factors between participants with and without depression were not significant. Although income level tended to be higher in the group without depression, the difference was marginally not significant (*p* value = 0.078).

### 3.2. Main Analysis 

Table 4 shows the associations of the owners’ depression symptoms with the PAS-M and covariates. The owners with higher PAS-M scores, indicating favorable attitudes toward their dogs, tended to have fewer depression symptoms (Model 1: OR = 0.95, 95% CI = 0.94–0.96). The direction and significance of the associations were maintained when the PAS-M variable was categorized as a dichotomous variable using the median value of 89. Participants with lower PAS-M scores had more depression symptoms than the others (Model 2: OR = 3.19, 95% CI = 2.28–4.47). The model using the continuous PAS-M variable had a better fit (Model 1, AIC = 851.19; Model 2, AIC = 865.28).

In terms of covariates, no association was significant. Although females tend to have higher depression symptoms than males, the association was marginally not significant in Model 1 (OR = 1.37, 95% CI = 0.97–1.96). Multicollinearity was not found in either model, as GVIF^1/(2*df)^ of all variables were less than 2 (Table S1).

### 3.3. Sensitivity Analysis 

Table 5 shows the results of the sensitivity analysis using the CESD-10 score as a continuous variable. The direction and significance of the associations between CESD-10 and PAS-M in Models 3 and 4 were the same as in Models 1 and 2. A significant negative linear association was found between PAS-M and the CESD-10 score (Model 3: β = −0.11, 95% CI = −0.15–−0.08), and participants with lower PAS-M scores had higher CESD-10 scores, indicating higher depression symptoms (β = 2.67, 95% CI = 1.79–3.54). The model using the continuous PAS-M variable had a better fit (Model 3, AIC = 4079.71; Model 4, AIC = 4093.99).

In terms of covariates, no association was significant, except for high-income level. In Model 4, participants in the highest tertile income group had lower CESD-10 scores than those in the lowest tertile group (β = −1.20, 95% CI = −2.31–−0.09). Although females tend to have higher CESD-10 scores than males, the association was marginally not significant in Model 3 (β = 0.86, 95% CI = −0.03–1.75). Multicollinearity was not found in either model as GVIF^1/(2*df)^ of all variables were less than 2 (Table S1).

## 4. Discussion

This study investigated the association between the depression symptoms of companion dog owners and their attitudes toward their dogs to explore whether the strength of the human–animal bond can modify the mental health benefits of dog ownership. The results showed that the owners with favorable attitudes toward their dogs had fewer depression symptoms. The significant association was robust, as models using different variable types (either continuous or categorical) for both depression symptoms and attitudes, and models with different covariates (Tables S2–S5) showed consistent results.

As a cross-sectional study, these results cannot determine a causal link between attitude and depression symptoms. Future studies are needed to shed light on the causal relationship, and direction should be investigated (i.e., whether the favorable attitude of the owner facilitates the health benefit of dog ownership, which in turn prevents depression, or whether the depression symptoms cause unfavorable attitudes toward the owners’ dogs). There are potential underlying explanations for both directions and either direction would have practical implications.

First, the owner’s attitude could affect the owner’s depression symptoms by enhancing the health benefit of ownership. Beetz et al. proposed that various health benefits of human–animal interactions (HAIs), including mental health, are mediated by increased oxytocin levels [24]. Their hypothesis was based on the finding that the HAIs could increase oxytocin levels [38,39], and both HAIs [11] and oxytocin [40,41] were associated with the health benefit. In their suggested causal link; however, the effect of HAI on oxytocin level could differ by owner. Although evidence is lacking, the oxytocin response has been found to be contingent on previous lifetime experience of dog ownership [23]. Considering that the previous experience of ownership or interactions with animals may have been associated with a positive attitude [25,42], the owner’s attitude might be associated with a different oxytocin response from the HAI. In summary, the effect of HAI on the oxytocin level could be associated with the owner’s attitude, which in turn affects the mental health benefit. The association between social capital and depression could be analogous. Social capital usually indicates social assets, including emotional resources available to individuals [43,44]. Social capital is categorized into cognitive and structural social capital [45]. Cognitive social capital typically refers to shared values, beliefs, and attitudes, such as neighborhood attachment; while structural social capital represents the presence of a network, such as social participation [44,45]. A review study showed that the preventive effects of cognitive social capital were more evident than those of structural social capital [46], and a study of adult twins reported that the negative association between cognitive social capital and depression was significant, even after adjusting for genetic and shared environmental factors [44]. Cognitive social capital corresponds to the owners’ attitudes toward their dogs, in that both are related to subjective belief and attitude rather than the “presence” of a relationship.

However, adopting current results without further investigation should be cautious. A previous study [47] found that either ownership or attachment did not show significant association with health in elderly population. A qualitative study [48] also suggested that the association between attachment and health benefit would be an inverse U shape, in that both extremely high and low attachment may be detrimental.

Future studies of the potential link between owners’ attitudes and depression symptoms could have practical implications, especially for animal-assisted intervention (AAI). Current AAI has faced criticism, mainly due to its unclear effects on human health [49,50]. Determining the different health effects of HAIs based on the owners’ attitudes suggest that the recipients’ attitudes should be considered before implementing an AAI program and sophisticated studies would help to clarify the health benefits. The importance of further studies was supported by a previous study that reported that people who had favorable attitudes toward animals tended to have positive perceptions and belief in AAI [51].

Alternatively, our results also suggested the potential effect of owners’ depression on their attitudes toward companion dogs. A causal link can be postulated from the findings of studies about parents. A recent review revealed that mothers with depression had a greater likelihood of negative parenting behaviors [52], and poor parenting practices could result from negative parental attitudes [53]. Likewise, dog owners with depression might feel a lower level of intimacy with their dogs, as indicated by the lower PAS-M score in this study. Investigating the causal link could imply a potential role of psychiatrist on companion animal care. For example, when people want to adopt stray dogs in animal shelters, the depression status of candidate owners could be examined, as candidate parents have done for children adoption.

In terms of covariates, none of the variables was significantly associated with depression in this study. These results were inconsistent with previous findings that socio-economic variables were associated with depression [54,55]. However, considering the direction of associations of covariates were consistent with previous studies, the relatively small sample size could make the non-significance.

There are several limitations to this study. First, the interpretation of the results is limited to young adults living in urban areas, and cannot be generalized to other age groups or those in rural areas. However, since there are no studies of the association between depression and attitude in young adults, our study has important implications. In addition, latent variables that should be considered in other age groups, such as the influence of parents or co-morbid chronic illness in the elderly, can be excluded, because we narrowed the range of the study population. Second, the self-reported measures we used could lead to information biases. For example, the self-reported pet attitude could exaggerate the “true” attitude, as a previous study reported similar patterns [56]. However, considering that the bias would be non-differential rather than differential, the bias has a limited effect on the direction of the association, making the results more conservative. Third, because the study population was employed by convenience sampling, they are not the representative general population in Seoul. For example, 86.5% of study participants graduated from universities, but an official survey with a representative sample in Seoul revealed that only 68.4% of residents aged 19 to 39 years old had a university degree in 2017. In addition, the prevalence of depression in the study population was too higher (51.1% and 55.1% for aged 19–29 and 30–39 years, respectively) than the general population in South Korea (9.5% and 7.8% for aged 19–29 and 30–39 years, respectively) [55]. Finally, as described throughout this manuscript, we could not determine the direction of the effect in this study due to its cross-sectional design. Further studies with longitudinal design and larger sample size are needed to elaborate on the direction and underlying mechanisms mediating the results. Sophisticated measurement could also profit from reliable results. For example, capturing stress level from employment could differentiate the risk effect of stress from the protective effect of economic security. Other attitude measures could also be employed. As PAS-M captures general attitude, measurement for the bond between an individual person and their individual animal would be necessary.

## 5. Conclusions 

In this study, we purposed to investigate the association between the depression symptoms of companion dog owners and their attitudes toward their dogs to explore whether the strength of the human–animal bond can modify the mental health benefits of dog ownership. Employing young adults whose major disease burden were depression in South Korea, we found a significant association between owners’ depression symptoms and less friendly attitudes toward their dogs. As a cross-sectional study with a convenient sample, the association cannot refer to a causal link. However, the robust results of this study with various models suggest that future longitudinal studies with a representative sample are necessary to investigate the potential causal link. If the follow-up studies confirmed the association, whichever the causal direction, its implications could be applied on AAT or animal care.

## Figures and Tables

**Table 1:** Variables used in this study and their re-categorization.

Category	Factors	Data Collected	Re-Categorization
Outcome variable	Depression symptom	CESD-10 (10 items)	Categorical With depression (score 11–30) Without depression (score 0–10)
Explanatory variable	Owners’ attitudes toward their dogs	PAS-M (18 items)	Continuous (score 1–126) Categorical High (higher than 89) Low (less or equal to 89)
Covariates	Age	Continuous (19–39)	Categorical 19–29 30–39
	Sex	Categorical Men Women	-
	Education	Categorical ≤Elementary school Middle school High school ≥University degree	Categorical ≤High school ≥University degree
	Income level	Continuous (monthly income)	Categorical Highest tertile Middle tertile Lowest tertile
	Marital status	Categorical Married Separated Bereaved Divorced Single	Categorical Married Currently single (separated/ bereaved/ divorced/ single)
	Employment	Categorical Employed Unemployed	-
	Family number	Continuous	Categorical One or two Three or more

Note: Nine variables were investigated in this study and the variables were re-categorized for the statistical analysis.

**Table 2:** The questions in the modified Pet Attitude Scale.

ID	Question	Note
1	I really like seeing pets enjoy their food	
2	My pet means more to me than any of my friends	
3	I would like to have a pet in my home	
4	Having pets is a waste of money	Reverse score
5	House pets add happiness to my life	
6	I feel that pets should always be kept outside	Reverse score
7	I spend time every day playing with my pet	
8	I have occasionally communicated with my pet and understood what it was trying to express	
9	The world would be a better place if people would stop spending so much time caring for their pets and started caring more for other human beings instead	
10	I like to feed animals out of my hand	
11	I love pets	
12	Animals belong in the wild or in zoos, but not in the home	Reverse score
13	If you keep pets in the house you can expect a lot of damage to furniture	Reverse score
14	I like house pets	
15	Pets are fun but it is not worth the trouble of owning one	Reverse score
16	I frequently talk to my pets	
17	I hate animals	Reverse score
18	You should treat your house pets with as much respect as you would a human member of your family	

Note: The Pet Attitude Scale was proposed by Templer et al. [30] and modified by Munsell et al. [29]. The questions were extracted from Anderson et al. [31] and translated into Korean for this study.

**Table 3:** General characteristics of the study population.

Variables	Levels	Total		With Depression		Without Depression		*p*-Value
		N	%	N	%	N	%	
Total		654	100	353	100	301	100	
Age	19–29	184	28.1	94	26.6	90	29.9	0.401
	30–39	470	71.9	259	73.4	211	70.1	
Sex	male	362	55.4	198	56.1	164	54.5	0.739
	female	292	44.6	155	43.9	137	45.5	
Education	≤elementary school	7	1.1	4	1.1	3	1.0	0.645
	middle school	4	0.6	2	0.6	2	0.7	
	high school	77	11.8	44	12.5	33	11.0	
	≥university degree	566	86.5	303	85.8	263	87.4	
Income	high	225	34.4	108	30.6	117	38.9	0.078
	middle	228	34.9	128	36.3	100	33.2	
	low	201	30.7	117	33.1	84	27.9	
Marital status	with spouse	324	49.5	176	49.9	148	49.2	0.923
	without	330	50.5	177	50.1	153	50.8	
Employment	employed	568	86.9	306	86.7	262	87.0	0.985
	unemployed	86	13.1	47	13.3	39	13.0	
Family size	one	31	4.7	15	4.2	16	5.3	0.579
	two	101	15.4	51	14.4	50	16.6	
	three or more	522	79.8	287	81.3	235	78.1	
Depression	yes	353	54.0					
	no	301	46.0					
		**Mean**	**S.D.**	**Mean**	**S.D.**	**Mean**	**S.D.**	***p*-Value**
PAS-M ^1^		89.57	13.7	85.71	13.5	94.1	12.6	< 0.001

Note: CESD-10 was used to measure owners’ depression symptoms (scores higher than 10 were categorized as having depression symptoms). The *p*-value was estimated using the Pearson chi-square test for categorical variables and t-test for the continuous variables (PAS-M). ^1^ PAS-M, modified Pet Attitude Scale.

**Table 4:** Association of owners’ depression symptoms (categorical variables) with the modified Pet Attitude Scale.

Explanatory Variables	Model 1		Model 2	
	OR	95% CI	OR	95% CI
PAS-M ^1^ (continuous)	0.95	0.94–0.96		
PAS-M ^1^				
high (>89)			Ref	–
low (≤89)			3.19	2.28–4.47
Age				
19–29	Ref	–	Ref	-
30–39	1.28	0.85–1.95	1.22	0.81–1.84
Sex				
male	Ref	–	Ref	–
female	1.37	0.97–1.96	1.24	0.88–1.75
Education				
high school or less	Ref	–	Ref	–
university of more	0.95	0.57–1.57	0.94	0.57–1.54
Income				
low tertile	Ref	–	Ref	–
middle tertile	0.88	0.58–1.34	0.85	0.56–1.29
high tertile	0.73	0.48–1.12	0.70	0.46–1.07
Marital status				
single	Ref	–	Ref	–
married	0.81	0.56–1.18	0.89	0.62–1.28
Employment				
unemployed	Ref	–	Ref	–
employed	0.94	0.58–1.53	0.93	0.57–1.49
Family size				
one or two	Ref	–	Ref	–
three or more	1.22	0.80–1.86	1.29	0.85–1.97
AIC	851.19		865.28	

Note: The CESD-10 was used to measure owners’ depression symptoms (scores higher than 10 were categorized as having depression symptoms). All models were adjusted for demographic and socioeconomic factors (age, sex, education level, income, marital status, and employment). ^1^ The modified Pet Attitude Scale (PAS-M) was included as a continuous variable in Model 1 and as a categorized variable in Model 2, based on the median value, which was 89.

**Table 5:** Association of owners’ depression symptoms (continuous variable) with the modified Pet Attitude Scale.

Explanatory Variables	Model 3		Model 4	
	β	95% CI	β	95% CI
PAS-M ^1^ (continuous)	−0.11	−0.15–−0.08		
PAS-M ^1^				
high (>89)			Ref	–
low (≤89)			2.67	1.79–3.54
Age				
19–29	Ref	–	Ref	–
30–39	0.41	−0.65–1.48	0.30	−0.78–1.38
Sex				
male	Ref	–	Ref	–
female	0.86	−0.03–1.75	0.61	−0.28–1.50
Education				
high school or less	Ref	–	Ref	–
university of more	−0.65	−1.93–0.64	−0.67	−1.97–0.63
Income				
low tertile	Ref	–	Ref	–
middle tertile	−0.48	−1.55–0.59	−0.57	−1.65–0.51
high tertile	−1.09	−2.18 – 0.01	−1.20	−2.31–−0.09
Marital status				
single	Ref	–	Ref	–
married	−0.86	−1.08–0.08	−0.68	−1.62–0.27
Employment				
unemployed	Ref	–	Ref	–
employed	−0.62	−1.86–0.63	−0.65	−1.91–0.61
Family size				
one or two	Ref	–	Ref	–
three or more	0.48	−0.62–1.57	0.61	−0.49–1.72
AIC	4079.71		4093.99	

Note: The CESD-10 was used to measure owners’ depression symptoms (scores were used as a continuous variable). All models were adjusted for demographic and socioeconomic factors (age, sex, education level, income, marital status, and employment). ^1^ The modified Pet Attitude Scale (PAS-M) was included as a continuous variable in Model 3, and a categorized variable in Model 4, based on the median value, which was 89.

## Supplementary Materials

The following are available online at https://www.mdpi.com/1660-4601/16/19/3567/s1, Table S1: Generalized variance inflation factors of each explanatory variable, Table S2: Robustness of the association between owner’s depression symptoms and the modified Pet Attitude Scale (Model 1), Table S3: Robustness of the association between owner’s depression symptom and the modified Pet Attitude Scale (Model 2), Table S4: Robustness of the association between owner’s depression symptom and the modified Pet Attitude Scale (Model 3), Table S5: Robustness of the association between owner’s depression symptom and the modified Pet Attitude Scale (Model 4).
