# Supplementary material for: Owners’ Attitudes toward Their Companion Dogs Are Associated with the Owners’ Depression Symptoms—An Exploratory Study in South Korea
Source: Int J Environ Res Public Health. 2019 Sep 24;16(19):3567. doi: 10.3390/ijerph16193567 (PMC6801548; doi:10.3390/ijerph16193567)
Supplement: Supplementary file 1 [file ijerph-16-03567-s001.zip › ijerph-583557-supplementary.pdf]

Table S1 Generalized variance inflation factors of each explanatory variable

Variables	GVIF	DF	$GVIF^{1/(2*df)}$
**Model 1**			
PAS-M (continuous)	1.133	1	1.065
Age	1.339	1	1.157
Sex	1.154	1	1.074
Education	1.115	1	1.056
Income	1.165	2	1.039
Marital status	1.300	1	1.140
Employment	1.018	1	1.009
Family size	1.156	1	1.075
**Model 2**			
PAS-M	1.092	1	1.045
Age	1.334	1	1.155
Sex	1.129	1	1.063
Education	1.111	1	1.054
Income	1.168	2	1.040
Marital status	1.276	1	1.130
Employment	1.018	1	1.009
Family size	1.157	1	1.076
**Model 3**			
PAS-M (continuous)	1.116	1	1.056
Age	1.324	1	1.151
Sex	1.137	1	1.066
Education	1.110	1	1.054
Income	1.170	2	1.040
Marital status	1.280	1	1.132
Employment	1.016	1	1.008
Family size	1.155	1	1.075
**Model 4**			
PAS-M	1.087	1	1.043
Age	1.328	1	1.152
Sex	1.114	1	1.055
Education	1.111	1	1.054
Income	1.171	2	1.040
Marital status	1.270	1	1.127
Employment	1.017	1	1.008
Family size	1.153	1	1.074

Table S2. Robustness of the association between owner's depression symptoms and the modified Pet Attitude Score (Model 1)

Explanatory variables	**Model 1**[1]		**Model S1.1**[2]		**Model S1.2**[3]	
	OR	95% CI	OR	95% CI	OR	95% CI
PAS-M	0.95	0.94 - 0.96	0.95	0.94 - 0.96	0.95	0.94 - 0.96
Age						
19-29	Ref	-			Ref	-
30-39	1.28	0.85 - 1.95			1.12	0.78 - 1.63
Sex						
male	Ref	-			Ref	-
female	1.37	0.97 - 1.96			1.38	0.98 - 1.96
Education						
high school or less	Ref	-				
university of more	0.95	0.57 - 1.57				
Income						
low tertile	Ref	-				
middle tertile	0.88	0.58 - 1.34				
high tertile	0.73	0.48 - 1.12				
Marital status						
single	Ref	-				
married	0.81	0.56 - 1.18				
Employment						
unemployed	Ref	-				
employed	0.94	0.58 - 1.53				
Family size						
one or two	Ref	-				
three or more	1.22	0.80 – 1.86				
AIC	851.19		842.8		843.39	

Note: CESD-10 was used to measure owners' depression symptoms (scores higher than 10 categorized as having depression symptoms), and the modified Pet Attitude Scale (PAS-M) was included as a continuous variable

[1] Model 1: Adjusted for demographic and socioeconomic factors (age, sex, education level, income, marital status, and employment; [2]Model S1.1: Univariate analysis; [3]Model S1.2: Adjust for demographic factors (age and sex)

Table S3. Robustness of the association between owner's depression symptom and the modified Pet Attitude Score (Model 2)

Explanatory variables		Model 2[1]		Model S2.1[2]		Model S2.2[3]	
		OR	95% CI	OR	95% CI	OR	95% CI
PAS-M*							
	high (>89)	Ref	-	Ref	-	Ref	-
	low (≤89)	3.19	2.28 - 4.47	3.15	2.29 - 4.35	3.28	2.36 - 4.58
Age							
	19-29	Ref	-			Ref	-
	30-39	1.22	0.81 - 1.84			1.09	0.76 - 1.58
Sex							
	male	Ref	-			Ref	-
	female	1.24	0.88 - 1.75			1.24	0.89 - 1.75
Education							
	high school or less	Ref	-				
	university of more	0.94	0.57 - 1.54				
Income							
	low tertile	Ref	-				
	middle tertile	0.85	0.56 - 1.29				
	high tertile	0.70	0.46 - 1.07				
Marital status							
	single	Ref	-				
	married	0.89	0.62 - 1.28				
Employment							
	unemployed	Ref	-				
	employed	0.93	0.57 - 1.49				
Family size							
	one or two	Ref	-				
	three or more	1.29	0.85 – 1.97				
AIC		865.28		855.20		857.57	

Note: CESD-10 was used to measure owners' depression symptoms (scores higher than 10 categorized as having depression symptoms), and the modified Pet Attitude Scale (PAS-M) was categorized based on the median value, which was 89

[1] Model 2: Adjusted for demographic and socioeconomic factors (age, sex, education level, income, marital status, and employment; [2]Model S2.1: Univariate analysis; [3]Model S2.2: Adjust for demographic factors (age and sex)

Table S4. Robustness of the association between owner's depression symptom and the modified Pet Attitude Score (Model 3)

Explanatory variables	**Model 3**[1]		**Model S3.1**[2]		**Model S3.2**[3]	
	β	95% CI	β	95% CI	β	95% CI
PAS-M*	-0.11	-0.15 – -0.08	-0.11	-0.14 – -0.08	-0.12	-0.15 – -0.09
Age						
19-29	Ref	-			Ref	-
30-39	0.41	-0.65 – 1.48			-0.22	-0.17 – 0.74
Sex						
male	Ref	-			Ref	-
female	0.86	-0.03 – 1.75			0.88	-0.01 - 1.78
Education						
high school or less	Ref	-				
university of more	-0.65	-1.93 – 0.64				
Income						
low tertile	Ref	-				
middle tertile	-0.48	-1.55 – 0.59				
high tertile	-1.09	-2.18 – 0.01				
Marital status						
single	Ref	-				
married	-0.86	-1.08 – 0.08				
Employment						
unemployed	Ref	-				
employed	-0.62	-1.86 – 0.63				
Family size						
one or two	Ref	-				
three or more	0.48	-0.62 – 1.57				
AIC	4079.71		4079.44		4078.84	

Note: CESD-10 was used to measure owners' depression symptoms and included as a continuous variable. The modified Pet Attitude Scale (PAS-M) was included as a continuous variable

[1] Model 3: Adjusted for demographic and socioeconomic factors (age, sex, education level, income, marital status, and employment; [2]Model S3.1: Univariate analysis; [3]Model S3.2: Adjust for demographic factors (age and sex)

Table S5. Robustness of the association between owner's depression symptom and the modified Pet Attitude Score (Model 4)

Explanatory variables	**Model 4**[1]		**Model S4.1**[2]		**Model S4.2**[3]	
	β	95% CI	β	95% CI	β	95% CI
PAS-M*						
high (>89)	Ref	-	Ref	-	Ref	-
low (≤89)	2.67	1.79 – 3.54	2.61	1.77 - 3.46	2.77	1.91 – 3.64
Age						
19-29	Ref	-			Ref	-
30-39	0.30	-0.78 – 1.38			-0.28	-1.25 – 0.69
Sex						
male	Ref	-			Ref	-
female	0.61	-0.28 – 1.50			0.64	-0.26 – 1.53
Education						
high school or less	Ref	-				
university of more	-0.67	-1.97 – 0.63				
Income						
low tertile	Ref	-				
middle tertile	-0.57	-1.65 – 0.51				
high tertile	-1.20	-2.31 – -0.09				
Marital status						
single	Ref	-				
married	-0.68	-1.62 – 0.27				
Employment						
unemployed	Ref	-				
employed	-0.65	-1.91 – 0.61				
Family size						
one or two	Ref	-				
three or more	0.61	-0.49 – 1.72				
AIC	4093.99		4091.36		4092.57	

Note: CESD-10 was used to measure owners' depression symptoms and included as a continuous variable. The modified Pet Attitude Scale (PAS-M) was categorized based on the median value, which was 89

[1] Model 4: Adjusted for demographic and socioeconomic factors (age, sex, education level, income, marital status, and employment; [2]Model S4.1: Univariate analysis; [3]Model S4.2: Adjust for demographic factors (age and sex)
